# Voluntary physical activity counteracts the proliferative tumor growth microenvironment created by adipose tissue via high‐fat diet feeding in female rats

**DOI:** 10.14814/phy2.13325

**Published:** 2017-07-04

**Authors:** Christopher F. Theriau, Michael K. Connor

**Affiliations:** ^1^ School of Kinesiology and Health Science York University Toronto ON Canada; ^2^ Muscle Health Research Centre York University Toronto ON Canada

**Keywords:** Adipokines, breast cancer, estrogen, obesity, physical activity

## Abstract

The adipokine secretion profile created from adipose tissue may represent the molecular mechanism behind the obesity‐breast cancer association. Two adipokines, adiponectin (ADIPO), and leptin (LEP), are altered with obesity and exert antagonistic effects on breast cancer proliferation. We set out to determine whether the adipose‐dependent tumor promoting growth environment created by a high‐fat diet (HFD) in female Sprague‐Dawley rats is altered compared to established responses in male rats and whether voluntary physical activity (PA) ameliorates any HFD‐dependent effects. We found that conditioned media (CM) created from the adipose tissue of female HFD‐fed rats increased the proliferation of MCF7 cells compared to those cells grown in CM prepared from lean adipose tissue. HFD‐CM inhibited AMPK and activated Akt signaling, decreased p27 phosphorylation at T198, reduced total p27 and AdiporR1 protein levels and promoted cell‐cycle entry. PA reversed the proliferative effects of HFD‐CM on MCF7 cells by preventing the effects of HFD on AMPK, Akt, p27 and AdipoR1, ultimately resulting in cell‐cycle withdrawal. Overexpressing AdipoR1 abolished the proliferative effects of the HFD‐CM on MCF7 cells and enhanced the anti‐proliferative effects PA on the HFD‐CM. Thus, PA represents a means to prevent deleterious obesity‐related alterations in tumor growth environment which are brought about by changes in adipokine secretion profile from adipose tissue in the presence of estrogen. Furthermore, although adipose produces hundreds of adipokines, the ADIPO:LEP ratio may serve to indicate the contribution of adipose in creating a tumor growth microenvironment.

## Introduction

Breast cancer is the most commonly diagnosed malignancy among women in the world. For almost 50 years there has existed a statistical link between adiposity and an increased risk of breast cancer (Sneddon et al. 1968). Several clinical and preclinical studies have demonstrated that increased adiposity is associated with increased cancer incidence, morbidity, poorer response to therapy and higher disease mortality (Sneddon et al. 1968; Calle et al. 2003; Parekh et al. 2012). While this association appears to be strong in postmenopausal women (Xia et al. 2014), the relationship between obesity and breast cancer in premenopausal women is far less consistent. Studies have reported that obesity in premenopausal women is inversely associated with breast cancer (van den Brandt et al. 2000; Michels et al. 2006), shows no association (Kaaks et al. 1998; Lahmann et al. 2004), or shows a positive association with disease development (Cecchini et al. 2012). This increased incidence of breast cancer in obese postmenopausal women has been suggested to be due, in part, to the direct effects of estrogen on the peripheral fat depots via aromatization of androgens (Catalano et al. 2003, 2004).

Adipose tissue has been identified as an active endocrine organ‐producing adipocyte‐derived factors, termed adipokines. These adipokines can act in an autocrine, endocrine and/or paracrine manner. While some adipokines are secreted from other tissues in the body, the vast majority are produced/secreted by white adipose tissue. Thus far, over 400 adipokines have been discovered and several have been shown to become dysregulated in obese individuals (Zhong et al. 2010). Adiponectin (ADIPO) and leptin (LEP) represent major potential contributors to the adipose‐dependent microenvironment. Both are among the most abundant adipokines produced/secreted, are altered by obesity and have documented cell‐cycle regulatory effects on breast cancer cells (Dieudonne et al. 2002, 2006; Jarde et al. 2009). LEP is predominately produced by white adipose tissue and its level in the peripheral circulation is directly proportional to BMI (Wauters et al. 2000). LEP activates several intracellular pathways implicated in breast carcinogenesis, including the phosphoinositide‐3/Akt kinase signaling pathway (Garofalo and Surmacz 2006; Jarde et al. 2009). LEP activates Akt, which phosphorylates p27 at T157, preventing both its nuclear accumulation and inhibition of cyclin E/cdk2, thereby leading to cell‐cycle entry (Dieudonne et al. 2002; Liang et al. 2002; Garofalo et al. 2006). Conversely, ADIPO production/secretion decreases with obesity and induces cell‐cycle exit by activating AMPK, which directly phosphorylates p27 at T198, increasing p27 stability and inducing G1 arrest (Dieudonne et al. 2006; Liang et al. 2007; Grossmann et al. 2008). This is mediated by ADIPO binding to its receptor Adiponectin receptor 1 (AdpoR1) which is also implicated in breast cancer (Dieudonne et al. 2006; Theriau et al. 2016). ADIPO‐dependent anti‐proliferative effects are abolished by siRNA knockdown of AdipoR1 (Grossmann et al. 2008; Nakayama et al. 2008). Decreased ADIPO signaling through AdipoR1 has been shown to be associated with higher tumor grade and poorer patient outcomes in breast cancer patients (Pfeiler et al. 2010). We have previously demonstrated that increasing AdipoR1 levels in breast cancer cells increases the cell‐cycle inhibitory effects of ADIPO, via AMPK signaling, and can counteract the antagonism of ADIPO by LEP (Theriau et al. 2016). Clinical studies in postmenopausal women also suggest that decreased ADIPO:LEP ratios, rather than the levels of each adipokine individually, are stronger predictors of breast cancer risk (Ollberding et al. 2013). In premenopausal women, this ADIPO:LEP breast cancer association is less clear.

A sedentary lifestyle is widely accepted as a major contributor to the increase in obesity and its associated disorders (Schrauwen and Westerterp 2000), suggesting that physical activity (PA) can potentially serve as an intervention for obesity‐associated effects on breast cancer (Enger et al. 2000; Bradley et al. 2008). Although there are some discrepancies for the role of obesity and breast cancer risk in pre‐ versus postmenopausal women, research shows that PA can lower the risk of breast cancer regardless of menopausal status (Bernstein et al. 1994; Carpenter et al. 1999). Obese and physically inactive breast cancer patients appear to be at an increased risk for both disease progression and cancer‐related mortality, regardless of menopausal status (Pichard et al. 2008; Renehan et al. 2008; Doyle et al. 2012). Higher recreational PA has been shown to be associated with a 30–60% reduction in all estrogen receptor subtypes in premenopausal women compared to sedentary women (Enger et al. 2000). In mice, voluntary PA increases the circulating ADIPO:LEP ratio, decreases breast cancer incidence compared to sedentary controls by decreasing pAkt^T473^ and increased pAMPK^T172^ and p27 within mammary carcinomas (Zhu et al. 2008, 2012; Thompson et al. 2010). In agreement, women who consumed a calorie restricted diet coupled with moderate PA experienced a 9.5% increase in ADIPO and a 40.1% decrease in LEP (Abbenhardt et al. 2013). Thus, there are clear positive effects of the volume of physical activity to breast cancer patient prognosis. However, the contribution of estrogen in these phenomena remains unclear.

In male rats, which have very low circulating estrogen levels, visceral adipose of HFD‐fed rats induced cell‐cycle entry in MCF7 cells by activating Akt, inhibiting AMPK, decreasing ADIPO:LEP ratio and repressed AdipoR1 and p27 expression (Theriau et al. 2016). These HFD‐dependent effects were abolished by both voluntary PA and increased expression of AdipoR1. Given some of the inconsistencies surrounding the role of estrogen in adipose‐dependent increases in breast cancer development, we set out to determine whether the adipose‐dependent tumor growth microenvironment created by an HFD in female Sprague‐Dawley rats is altered compared to established responses in male rats and whether voluntary physical PA or increased expression of AdipoR1 ameliorates these effects. We show that the ADIPO:LEP ratio is decreased in the circulation of HFD‐fed female rats. This altered adipokine secretion profile caused a decrease in pAMPK^T172^, p27^T198^, AdipoR1, and an increase in pAkt^T308^ in MCF7 cells grown in conditioned media (CM) prepared from the adipose of HFD animals. This HFD‐CM lead to cell‐cycle entry by increasing the number of MCF7 cells in S‐phase while decreasing the number in G0/G1. Voluntary PA increased the ADIPO:LEP ratio compared to HFD sedentary animals, abolishing the effects of HFD‐CM on MCF7 proliferation. Overexpressing AdipoR1 in MCF7 cells counteracted the effects of the HFD‐CM. The adipokine levels in the CM produced by adipose from females was very different than that shown in previous work in male animals (Theriau et al. 2016). This altered HFD‐CM secretome blunted MCF7 proliferation compared to males (Theriau et al. 2016). These results highlight the importance of PA and stabilizing AdipoR1 signaling in order to overcome the positive tumor growth microenvironment created by obesity regardless of whether estrogen is present.

## Methods

### Animals

All animal experiments were approved by the York University Animal Care Committee in accordance with Canadian Council for Animal Care guidelines. Thirty female Sprague‐Dawley rats (7 weeks of age) were purchased from Charles River Laboratories (Montreal, QC, Canada) and were singly housed in standard clear, plastic cages. All animals had a 7‐day habituation period to a 12 h light‐dark cycle (lights on at 0600) in a temperature (22°C) and humidity (50–60%) controlled room. This age of animals was chosen as it is consistent with previously published data (Zhu et al. 2008, 2012; Thompson et al. 2010; Theriau et al. 2016) and ensured that the animals would maintain running distances throughout the entire protocol.

After acclimatization, animals were randomly selected and given free access to a running wheel (wheel circumference, 106 cm; Harvard Apparatus, Holliston, MA) within the cage. A magnetic counter mounted to each wheel detected the revolutions and distance run was calculated every 24 h. Animals acclimated to the wheels for 7 days and were subsequently divided into two groups: chow diet (CD; *n* = 10) and high‐fat diet (HFD; *n* = 20) with both groups given access to food and water (ad libitum). The CD (no. 5012 Lab Chows, Ralston Purina, St. Louis, MO) had caloric make‐up of 14% fat, 54% carbohydrate, 32% protein (3.02 calories/g). The HFD (Harlan Laboratories, Madison, WI) had a caloric breakdown of 60% fat, 21% carbohydrate, 18% protein (5.1 calories/g). Daily caloric intake for all animals was calculated. HFD and CD‐fed animals were further subdivided into sedentary and physical activity (PA) groups designated as chow diet‐sedentary (CD; *n* = 6), chow diet‐high physical activity (CD + HPA; >12.5 km/day; *n* = 4), HFD‐sedentary (HFD; *n* = 8). Animals in the HFD‐PA group were further subdivided depending on average wheel running distances into animals that run <12.5 km/day (*n* = 4) classified as “low physical activity” group (HFD + LPA) while animals that run more than 12.5 km/day (*n* = 8) were classed as “high physical activity” (HFD + HPA) to determine if volume effects of physical activity were evident. Our physical activity cutoff of 12.5 km/day has also been used previously with female Sprague‐Dawley rats and wheel running to create a low and high PA groups (Tokuyama et al. 1982; Gollisch et al. 2009). Although these cutoffs have been previously established in the literature, they may also be attributed to an animal's predisposition to PA. Food intake and running distances were measured each day and body weight was measured three times per week. After a 6‐week protocol, animals were anesthetized using isoflurane and tissues removed.

### Tissue collection and conditioned media

Visceral adipose tissue (periovarian, perirenal, retroperitoneal) was quickly removed from all animals and cultured as previously described (Sutherland et al. 2009; Theriau et al. 2016). In order to ensure that our mass:volume preparation of CM was consistent among preparations across groups, we diluted 10–15 mg sections of adipose 30:1 in RIPA buffer for protein extraction. Equal volumes of lysate (25 *μ*L) were subjected to SDS‐PAGE, using 12% gels and membranes were probed for total Akt and *β*‐actin to evaluate equivalency of specific protein content between preparations. Weights of all tissues collected were measured and normalized per 100 g of body weight. The sequence in which rats were killed was randomized across groups so as to minimize the likelihood that order effects would masquerade as treatment‐associated effects.

At the time of killing, the gastrocnemius, soleus and tibialis anterior were immediately excised, weighed, frozen, and stored at −84°C for future analyses. Also, 2–3 mL of blood was taken at the time of killing, left for 30 min on ice, and subsequently centrifuged. The serum was then extracted, frozen and stored at −84°C for future analyses.

### Cytochrome c oxidase activity assay

In order to confirm that the physical activity protocol elicited a training effect, we measured cytochrome c oxidase (COX) activity in mixed gastrocnemius muscles. COX activity was determined as previously described (Theriau et al. 2016). Briefly, cross‐sections of mixed gastrocnemius muscles (from the mid‐section of the muscle belly) weighing roughly 20–30 mg were diluted 80‐fold (sedentary) or 160‐fold (PA) in extraction buffer (100 mmol/L Na‐K‐Phosphate, 2 mmol/L EDTA, pH 7.2). Muscle extracts were prepared by homogenization with metal beads in a magnetic homogenizer (Mixer Mill MM 400, Retsch, Haan, Germany). These homogenates were then used for the analyses of the maximum rate of oxidation of fully reduced cytochrome c at 30°C as indicated by changes in absorbance (550 nm).

### Conditioned media/serum adipokine and estradiol measurement

The levels of ADIPO, LEP and estradiol (E2) produced and secreted into the co‐culture media by the adipose tissue as well as in the serum at the time of killing was determined, using rat adiponectin sandwich ELISA kit (BioVision, Milpitas, CA), mouse/rat leptin quantikine sandwich ELISA kit (R&D Systems, Minneapolis, MN), and rat estradiol competitive‐binding sandwich ELISA kit (Alpco Diagnostic, Salem, NH), as per manufacturer instructions. Aliquots of CM were diluted 50‐fold (HFD) and 100‐fold (PA and CD) for ADIPO ELISAs, fivefold (HFD) or undiluted (PA and CD) for LEP ELISAs and undiluted for E2 ELISAs. Aliquots of serum were diluted 1000‐fold for all samples for ADIPO ELISAs, fivefold (HFD and HFD + PA) or undiluted (PA and PA + HPA) for LEP ELISAs and E2 ELISAs. The levels of each adipokine were calculated in ng/mL values and ADIPO/LEP converted to nmol/L values for stoichiometric comparison. The levels of E2 were calculated in pg/mL values. We utilized the ADIPO:LEP ratio as a representation of the adipose‐derived microenvironment as both have been shown to have cell‐cycle regulatory effects on breast cancer cells and change predictably and reliably with obesity. This ratio allows for a prediction of the nature of the tumor growth microenvironment created by adipose tissue which may end up being of use in a clinical setting.

### Cell culture

MCF7 cells were purchased from the American Tissue type Culture Collection (ATCC, Manassas, VA) and were maintained in AMEM, 10% FBS, 2% antimicotic/antibiotic, 1 mmol/L sodium pyruvate, nonessential amino acids, and 10 *μ*g/mL insulin from human pancreas at 37°C and 5% CO_2_.

MCF7 cells were transfected with an AdipoR1 overexpressing plasmid vector as previously described (Theriau et al. 2016). Mock transfected (MockT) MCF7 cells and stably transfected AdipoR1 (AdipoR1‐T) cells were seeded in six well plates with AMEM for 24 h. At 70% confluence, cells were washed with PBS and incubated with CM produced from adipocytes for 24 h. MCF7 cells grown in AMEM supplemented with 10% FBS served as untreated controls (UT).

### Immunoblotting

The effects of adipokines on specific cellular proteins were measured using standard SDS‐PAGE protocols utilizing 12% polyacrylamide gels. Proteins (25 *μ*g) were transferred to PVDF membranes (Bio‐rad, Mississauga, ON, Canada), blocked for 2 h in 10% skim milk and subsequently incubated overnight with primary antibodies: p27^Kip1^ (BD Biosciences); p27^T198^ (R&D Systems); pAkt^T308^, Akt, pAMPK^T172^ , and AMPK (Cell Signaling); AdipoR1 (Santa Cruz Biotech, Santa Cruz, CA) and *β*‐actin (Abcam, Cambridge, MA). Anti‐mouse, anti‐rabbit (Promega, Madison, WI) and anti‐goat (Santa Cruz) horseradish peroxidase secondary antibodies were used to visualize proteins using Immobilon‐enhanced chemiluminescence substrate (Millipore, Whitby, ON, Canada) and detected/quantified on a Kodak In Vivo Pro imaging system (Marketlink Scientific, Burlington, ON, Canada).

### Cell‐cycle analyses

MCF7 cells isolated from 6‐well plates were trypsinized, washed in cold PBS and fixed by drop wise addition of ice‐cold 70% ethanol. Cells were washed twice in PBS, resuspended in a propidium iodide/RNAse solution and subjected to FACS analyses (Gallios Flow Cytometer, Beckman Coulter Mississauga, Canada). Cell‐cycle profiles were determined, using Mod‐fit software (Verity Software House, Topsham, ME), by fitting curves to profiles and measuring the areas under the curve to determine relative numbers of cells in G1, S, and G2/M phases.

### Statistical analyses

All values are expressed as mean ± SEM of 5 to 8 separate experiments (as indicated) and statistical analyses were performed, using a one‐way ANOVA with Tukey's post hoc tests conducted when significant main effects were found. Individual *t*‐tests were used to identify differences in FACS analysis between groups. Group means were considered to be significantly different when *P* ≤ 0.05.

## Results

### HFD‐dependent increases in adiposity are prevented by PA

**Figure 1:**
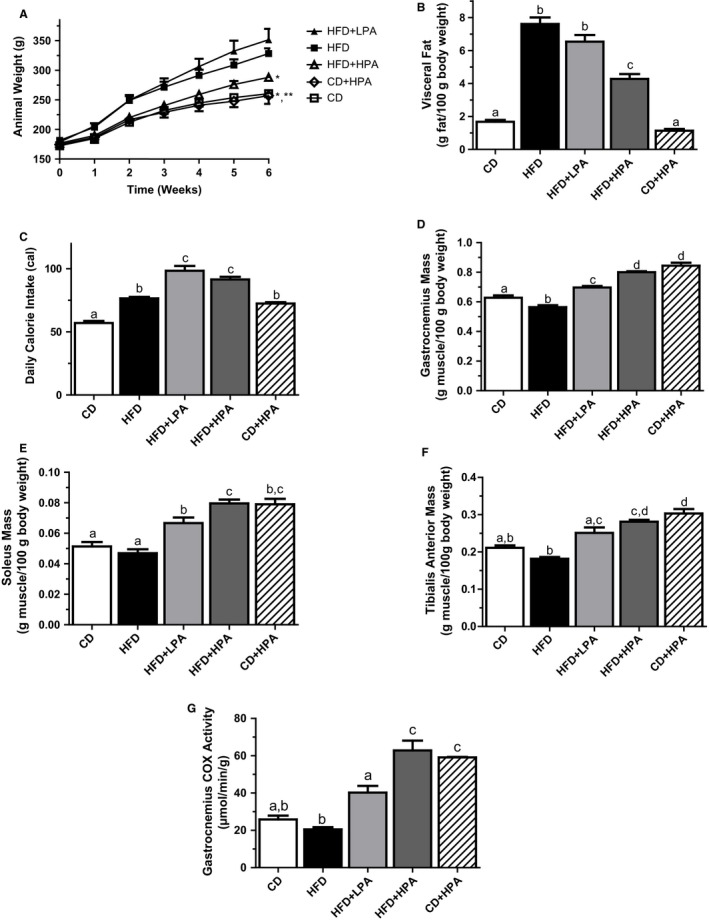
High‐fat diet increases total visceral fat and is ameliorated with PA. Body mass changes over the 6‐week protocol (A). Body weight adjusted total visceral fat mass in CD (open bar), HFD (black bar), HFD + LPA (light grey bar), HFD + HPA (dark gray bar) and CD + LPA (hatched bar) animals (B). Body weight adjusted muscle mass of the gastrocnemius (C), soleus (D) and tibialis anterior (E) muscles. Physical activity alters cytochrome C oxidase enzyme activity in the gastrocnemius muscles of CD, HFD, HFD + LPA, HFD + HPA and CD + LPA animals (F). *In A indicates different from HFD and HFD + LPA animals, **Indicates different from all other groups (*P* < 0.05). Different letters (B–F) indicate groups that are significantly different from each other (*P* < 0.05, *n* = 6/group). HFD, high‐fat diet; CD, chow diet; HFD + LPA, high‐fat diet + low physical activity; HFD + HPA, high‐fat diet + high physical activity; CD + LPA, chow diet + low physical activity.

HFD‐fed animals demonstrated a 26% higher total body mass compared to CD‐fed sedentary animals (328.±21.3 vs. 260.4 ± 10.3, respectively; Fig. 1A). This increased total body weight was mirrored by a 4.5‐fold increase total relative visceral fat mass (7.6 ± 0.97 vs. 1.68 ± 0.23 g/100 g body weight; Fig. 1B). The HFD sedentary animals also had an increased overall daily calorie intake compared to CD sedentary animals (76.6 ± 2.8 vs. 57.1 ± 3.2 cal/day; Fig. 1C), indicating that HFD animals were subjected to both increased fat and increased calorie ingestion. The difference in total body and total relative visceral weights between HFD and CD sedentary animals suggested that the HFD‐induced obesity in the animals.

HPA counteracted the HFD‐dependent increase in visceral adiposity as the HFD + HPA animals were 13% lighter than their sedentary counterparts (287.9 ± 15.8 g vs. 328.1 ± 21.3 g; Fig. 1A). Consistent with these results we observed a 44% decrease in total visceral fat mass in HFD + HPA compared to HFD sedentary animals (Fig. 1B). In contrast, HFD + LPA animals showed no difference in total body weight or specific visceral fat mass compared to their sedentary counterparts (Fig 1A and B). This may be due to the HFD + LPA group having a 72% higher daily calorie intake compared to HFD sedentary animals (98.5 ± 7.5 vs. 57.1 ± 3.2 cal/day, respectively; Fig. 1C). CD + HPA animals were 22% lighter and had 85% less total visceral fat mass compared to HFD sedentary animals (Fig. 1A and B). The CD + HPA and HFD + HPA animals ran similar distances (16.3 ± 2.3 vs. 18.2 ± 2.3 km/day) and both groups were found to have run more than the HFD + LPA group (9.6 ± 1.1 km/day).

In order to confirm that our PA protocol induced a specific aerobic training effect beyond that displayed by the decreases in body mass/adiposity, we looked for changes in the weights of the gastrocnemius, soleus and tibialis anterior muscles. PA increased gastrocnemius, soleus and tibialis anterior relative weights in both HFD + LPA and HFD + HPA animals compared to their sedentary counterparts (Fig. 1D–F). LPA and HPA increased muscle mass by an average of 29 ± 4% and 47 ± 3%, respectively, above HFD animals. This volume‐dependent effect of PA was evidenced by changes in oxidative enzyme capacities of mixed gastrocnemius muscles (Fig. 1G). LPA increased mixed gastrocnemius COX activity by 2.0‐fold while HPA further increased COX activity to levels that were 3.1‐fold above those in sedentary HFD‐fed animals.

### ADIPO:LEP ratio is decreased in the serum of HFD‐fed animals and this is prevented by PA

**Figure 2:**
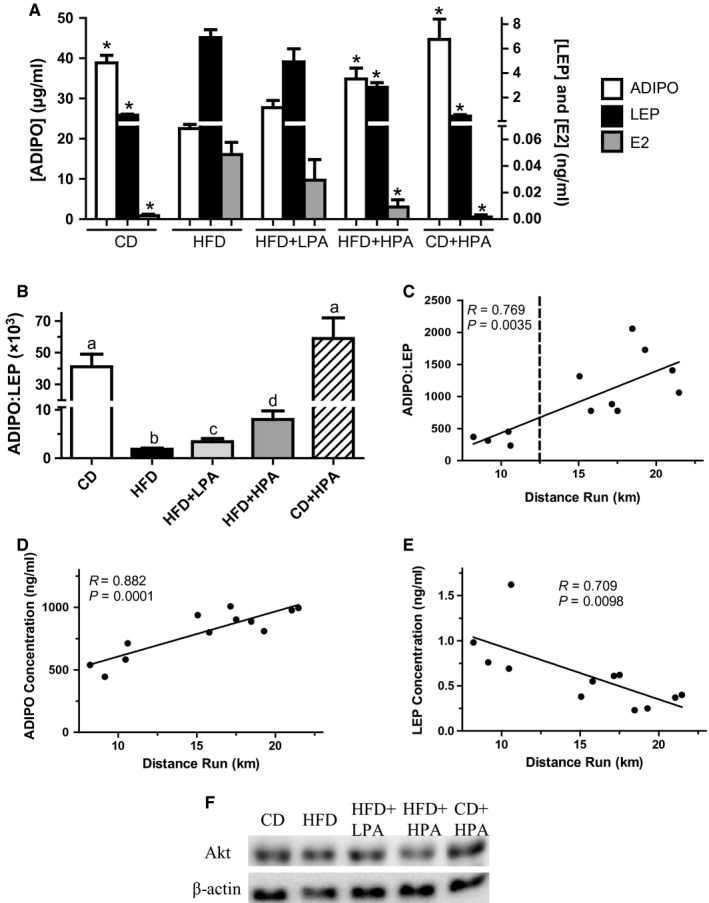
ADIPO is decreased, LEP and E2 increased in serum of HFD animals and reversed with HPA. Circulating serum ADIPO (open bar; *μ*g/mL), LEP (closed bar; ng/mL) and E2 (gray bar; ng/mL) concentrations at time of killing (A). Circulating serum ADIPO:LEP ratio at time of killing in CD (open bar), HFD (black bar), HFD + LPA (light gray bar), HFD + HPA (dark gray bar) and CD + LPA (hatched bar) animals at time of killing (B). Plotting of ADIPO:LEP ratio in CM versus daily km run (C). Dotted line indicates divider between HPA and LPA groups. Western blots showing levels of Akt and *β*‐actin in adipose tissues from the indicated groups (D). *In A indicates different from HFD (*P* < 0.05). Different letters (B) indicate groups that are significantly different from each other (*P* < 0.05, *n* = 6/group). HFD, high‐fat diet; CD, chow diet; HFD + LPA, high‐fat diet + low physical activity; HFD + HPA, high‐fat diet + high physical activity; CD + LPA, chow diet + low physical activity; ADIPO, adiponectin; LEP, leptin.

Serum ADIPO, LEP and E2 levels were determined at the time of sacrifice in order to determine if serum E2 levels correlated with any alterations in ADIPO and LEP due to HFD or PA. Serum E2 and LEP were both elevated by HFD and these increases were mitigated by diet (CD) and HPA but not LPA (Fig. 2A). The CD‐fed animals were found to have 92% lower LEP (6.90 ± 1.61 vs. 0.55 ± 0.17 ng/mL), 95% lower E2 (57.2 ± 9.2 vs. 2.6 ± 2.0 pg/mL) compared to HFD‐fed animals (Fig. 2A). Although the mean values for E2 and LEP were 29% and 47% lower in HFD + LPA than HFD sedentary animals, respectively, they were not different statistically. As a result of these ADIPO and LEP changes, there were corresponding changes in the ADIPO:LEP ratio (Fig. 2B). HFD caused a 97% decrease in the ADIPO:LEP ratio compared to CD animals (1854 ± 602 vs. 41,189 ± 15,901). LPA increased the ADIPO:LEP ratio 1.8‐fold while HPA increased this ratio by 4.3‐fold compared to that in HFD sedentary animals (Fig. 2B).

### HFD decreases the ADIPO:LEP ratio in adipose‐derived CM which is prevented by PA

Visceral adipose tissue depots were excised from animals in each of the experimental groups and used to prepare CM. Similar to serum, HFD‐CM showed a decreased ADIPO:LEP ratio compared to CD‐CM (128:1 vs. 2051:1; Table 1). This decreased ratio was brought about by an increased ADIPO and a decreased LEP in HFD‐CM. Compared to HFD‐CM, HFD + LPA‐CM had similar ADIPO but lower LEP levels, resulting in an overall increase in ADIPO:LEP ratio (128:1 vs. 341:1; Table 1). HFD + HPA‐CM had higher levels of ADIPO (889.6 ± 82.4 ng/mL) and lower levels of LEP (0.43 ± 0.15 ng/mL) than the HFD‐CM (ADIPO: 438.7 ± 71.5 ng/mL, LEP: 1.85 ± 0.21 ng/mL) with a resultant higher ADIPO:LEP ratio (128:1 vs. 1250:1). A linear relationship between the distance run and the ratio of ADIPO:LEP in the CM was found, suggesting that volume of PA is a major contributor regulating adipokine secretion from adipose tissue (*m* = 96.47 ± 25.47, *R* = 0.769, *P* = 0.0035; Fig. 2C). Individually, ADIPO was found to have a positive linear relationship with distance run (*m* = 36.06 ± 6.11, *R* = 0.882, *P* = 0.0001; Fig. 2D) while LEP has the opposite inverse relationship (*m* = −0.058 ± 0.018, *R* = 0.709, *P* = 0.0098). The levels of E2 were found to be no different in the CM among all groups (Table 1). Comparing the slopes of ADIPO:LEP and distance run between this study using female rats (*m* = 96.47 ± 25.47) and previously published data in males (*m* = 85.94 ± 21.83) (Theriau et al. 2016), we found no difference in the slopes of the linear regressions between the two studies despite a much greater distance run by the female animals in this study (*F* = 0.033, *P* = 0.86).

**Table 1:** ADIPO:LEP ratio for adipose‐derived conditioned media

Group	ADIPO (ng/mL)	LEP (ng/mL)	ADIPO:LEP	Estradiol (pg/mL)
CD	1166.0 ± 169.0^a^^,^ b	0.31 ± 0.09^a^^,^ b	2051.0 ± 336.3^a^^,^ b	42.2 ± 3.0
HFD	438.7 ± 71.5	1.85 ± 0.21	128.8 ± 28.2	46.8 ± 4.0
HFD + LPA	569.4 ± 111.2	0.96 ± 0.44^a^	341.2 ± 91.1^a^	49.8 ± 7.2
HFD + HPA	889.6 ± 82.4^a^	0.43 ± 0.15^a^^,^ b	1250.0 ± 467.5^a^^,^ b	48.9 ± 4.7
CD + HPA	1692.0 ± 584.7^a^^,^ b	0.39 ± 0.18^a^^,^ b	2398.0 ± 459.0^a^^,^ b	51.4 ± 12.2

HFD, high‐fat diet; CD, chow diet; HFD + LPA, high‐fat diet + low physical activity; HFD + HPA, high‐fat diet + high physical activity; CD + LPA, chow diet + low physical activity; ADIPO, adiponectin; LEP, leptin.

a Indicates significantly different from HFD.

b Indicates significantly different from HFD + LPA (*P* < 0.05, *n* = 6/group).

### Changes in adipokine secretion bring about diet and PA‐dependent effects on MockT MCF7 protein expression and signaling

**Figure 3:**
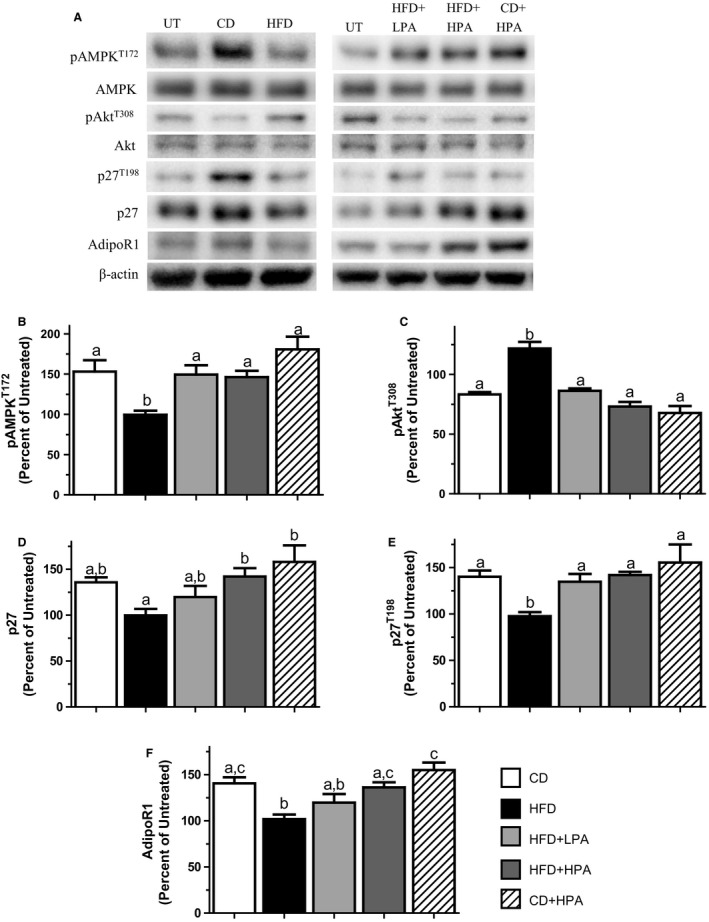
Physical activity abolishes the effect of an HFD on adipose‐dependent tumor growth microenvironment in MockT MCF7 cells. Representative western blots for selected proteins showing the effects of treatment with CM prepared from CD (open bar), HFD (black bar), HFD + LPA (light gray bar), HFD + HPA (dark gray bar) and CD + LPA (hatched bar) animals on MockT MCF7 cells (A). Graphical representations of multiple experiments showing the effects of CM on pAMPK^T^ ^172^ (B), pAKT^T^ ^308^ (C), p27 (D), p27^T198^ (E) and AdipoR1 (F) protein levels. *β*‐actin was used as a loading control. Different letters indicate groups that are significantly different from each other (*P* < 0.05, *n* = 6/group). HFD, high‐fat diet; CD, chow diet; HFD + LPA, high‐fat diet + low physical activity; HFD + HPA, high‐fat diet + high physical activity; CD + LPA, chow diet + low physical activity.

In order to verify that any effects observed between groups were not due to experimental design artifacts, we conducted western blot analyses using proteins isolated from the adipose tissue used in our CM preparations and measured the levels of Akt and *β*‐actin proteins to ensure equal protein contents among groups (Fig. 2F). We found no consistent differences between groups, supporting the notion that the CM was not subjected to any preparation artefacts. HFD‐CM decreased pAMPK^T172^, p27^T198^ and AdipoR1 while increasing pAkt^T308^ levels compared to CD‐CM‐treated MockT MCF7 cells (Fig. 3A–F). No difference was seen between HFD‐CM and CD‐CM in p27 protein levels (Fig. 3D). Interestingly, HFD‐CM appears to elicit the same effects on MCF7 cell protein expression as seen in UT cells (Fig. 3A). There were no changes evident in total AMPK and Akt.

Voluntary PA elicited a volume‐dependent response counteracting the effects of HFD. HFD + LPA‐CM was found to increase pAMPK^T172^ and p27^T198^ while decreasing pAkt^T308^ compared to HFD‐CM‐treated cells (Fig. 3A–C and E). The greatest effect of PA was found in HFD + HPA‐CM‐treated MCF7 cells as illustrated by increases in pAMPK^T172^, p27^T198^ and AdpoR1 by 47%, 46% and 33%, compared to HFD‐CM‐treated cells, respectively (Fig. 3A, B, D and F). In addition, pAkt^T308^ was decreased by 40% compared to HFD‐CM‐treated MCF7 cells (Fig. 3A and C). Both HFD + LPA‐CM and HPA‐CM had similar effects on all MCF7 proteins evaluated compared to CD‐CM (Fig. 3A–F). This suggests that at any level of voluntary PA, MCF7 protein expression was similar, abolishing the effect of HFD‐CM by increasing pAMPK^T172^ and p27^T198^ while decreasing pAkt^T308^. No changes in total AMPK and Akt were evident.

### AdipoR1 overexpression ameliorated the effects of the HFD‐CM and further enhanced the effects of PA in MCF7 cells

**Figure 4:**
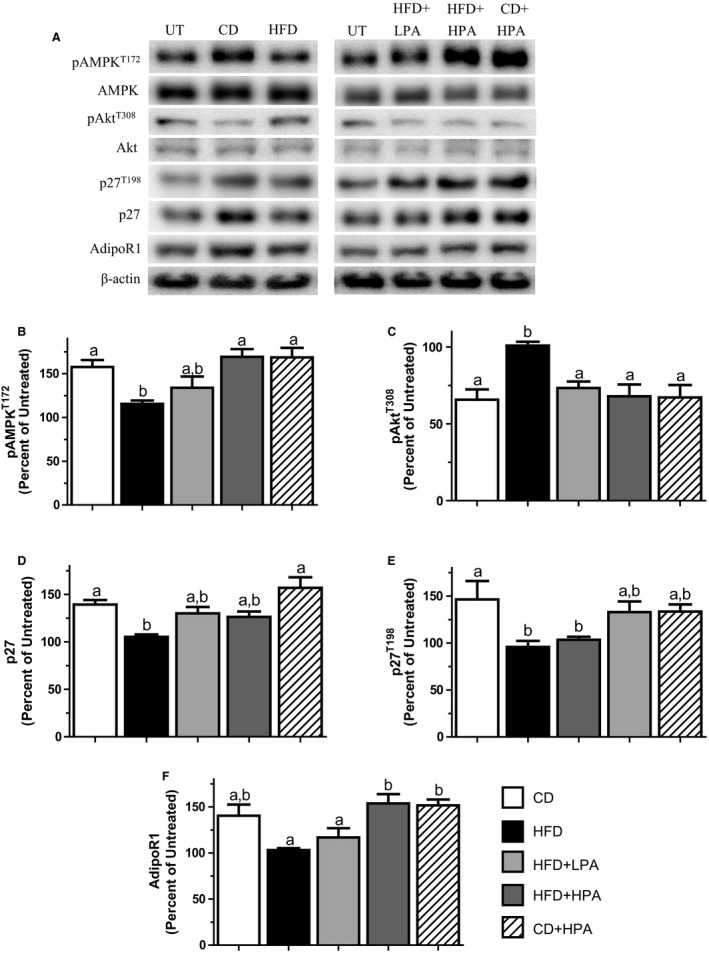
Overexpression of AdipoR1 can counteract the effects of HFD and accentuates the effects of PA. Representative western blots for selected proteins showing the effects of treatment with CM prepared from CD (open bar), HFD (black bar), HFD + LPA (light gray bar), HFD + HPA (dark gray bar) and CD + LPA (hatched bar) animals on AdipoR1 transfected (AdipoR1‐T) MCF7 cells (A). Graphical representations of multiple experiments showing the effects of CM on pAMPK^T^ ^172^ (B), pAKT^T^ ^308^ (C), p27 (D), p27^T198^ (E) and AdipoR1 (F) protein levels. *β*‐actin was used as a loading control. Different letters indicate groups that are significantly different from each other (*P* < 0.05, *n* = 6/group). HFD, high‐fat diet; CD, chow diet; HFD + LPA, high‐fat diet + low physical activity; HFD + HPA, high‐fat diet + high physical activity; CD + LPA, chow diet + low physical activity.

We next determined whether AdipoR1 overexpression could alter the effects of HFD‐CM. We have previously shown that our AdipoR1‐T MCF7 cells display a 2.7‐fold increase in AdipoR1 protein compared to MockT MCF7 cells (Theriau et al. 2016). MCF7 cells overexpressing AdipoR1 were used to determine any absolute/synergistic effects of augmented AdipoR1 signaling in MCF7 cell cycle regulation. HFD‐CM decreased pAMPK^T172^, p27 and p27^T198^ while increasing pAkt^T308^ compared to CD‐CM cells (Fig. 4A–E). No difference was found between AdipoR1 in HFD‐CM and CD‐CM‐treated AdipoR1‐T MCF7 cells (Fig. 4A and F), suggesting that AdipoR1 was constitutively overexpressed across treatment groups. Similar to MockT cells, HFD‐CM‐treated cells showed no difference between all proteins measured compared to UT cells. No changes in total AMPK and Akt were evident.

A volume‐dependent effect of PA was also evident in AdpoR1‐T cells. HFD + LPA‐CM was found to elicit the same effects as HFD‐CM on cells for all proteins except for pAkt^T308^ (Fig. 4A and C). In contrast, HFD + HPA‐CM increased pAMPK^T172^ (Fig. 4A and B) and AdipoR1 (Fig. 4A and F) by 47% and 50%, respectively compared to HFD‐CM‐treated cells while decreasing pAkt^T308^ by 33% (Fig. 4A and C) compared to HFD‐CM. Overall, AdipoR1 overexpression increased the levels of pAMPK^T172^, p27 and p27^T198^ in all treatment groups compared to MockT cells (Fig. 3A vs 4A). In fact, even though HFD‐CM elicited effects on the proteins measured, the expression levels were similar to those in MockT MCF7 cells grown in CD‐CM. These results highlight the importance of available AdipoR1 and indicate if the available binding sites for ADIPO can be increased; it is possible to override the obesity‐dependent cell‐cycle control regardless of the external growth environment.

### CM‐induced changes in MCF7 protein expression leads to overall cell‐cycle effects

**Figure 5:**
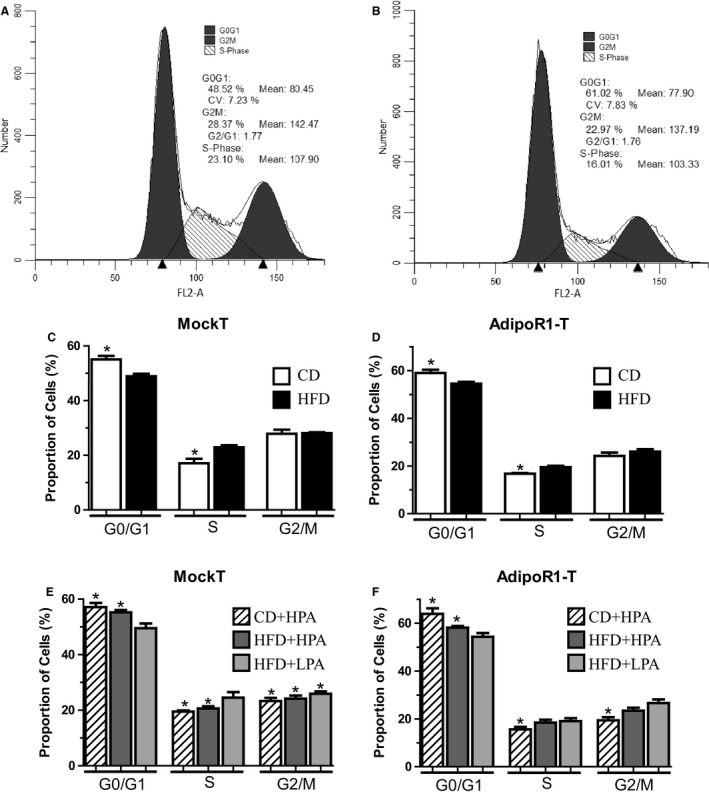
Adipose‐dependent growth environment causes cell‐cycle changes in CM experiments. Typical cell‐cycle profiles in MockT MCF7 cells (A) and stably transfected AdipoR1 overexpressing MCF7 cells treated with HFD‐CM (B). Graphical representation of multiple cell‐cycle profile experiments observing effects of diet on CM effects in CD (open bar), HFD (black bar) animals in MockT MCF7 cells (C) and in AdipoR1‐T MCF7 cells (D). Graphical representation of multiple cell‐cycle profiles showing the effects of exercise and diet CD + LPA (hatched bar), HFD + HPA (dark gray bar) and HFD + LPA (light gray bar) on MockT MCF7 cells (E) and MCF7 cells stably overexpressing AdipoR1 (F). *Indicate groups that are significantly different from HFD‐treated cells (*P* < 0.05, *n* = 6/group). HFD, high‐fat diet; CD, chow diet; HFD + LPA, high‐fat diet + low physical activity; HFD + HPA, high‐fat diet + high physical activity; CD + LPA, chow diet + low physical activity.

We next wanted to determine whether the diet and PA‐induced changes in CM‐treated MCF7 cell protein expression elicited overall cell‐cycle effects. Cell‐cycle status in MockT and AdipoR1‐T was determined, using propidium iodide staining and computational analyses (Fig. 5A and B). Cells that were exposed to HFD‐CM showed a 13% decrease in the number of cells in G1/G0 (55% vs. 48%) and a 26% increase in the number of cells in S‐phase (17% vs. 23%) compared to MockT MCF7 cells cultured in CD‐CM (Fig. 5C). AdipoR1 overexpression decreased the HFD‐dependent effects observed in MockT cells as HFD‐CM was found to cause only a 7% decrease in G0/G1 cells and an 11% increase in S‐phase cells compared to CD‐CM‐treated cells (Fig. 5D).

Increasing the expression of AdipoR1 increased the percentage of cells in G0/G1 and decreased the number of cells in S‐phase in both CD‐CM and HFD‐CM when compared to MockT‐treated cells (Fig. 5C vs. D). AdipoR1 overexpression increased the number of cells in G0/G1 by 12% (54% vs. 48%) and decreased the number of cells in S‐phase by 18% (23% vs. 19%) compared to MockT cells when exposed to HFD‐CM (Fig. 5C vs. D). This result agrees with previously published data in male rats (Theriau et al. 2016), as the effect of HFD‐CM is blunted in AdipoR1‐T MCF7 cells, stressing the potential protective effects of increasing AdipoR1 protein expression in the breast cancers of obese patients.

We also wanted to evaluate whether voluntary PA could counteract the HFD‐dependent overall cell‐cycle effects on MCF7 cells. We found a volume‐dependent effect of PA in MockT MCF7 cells. The HFD + HPA‐CM caused a 13% increase in G1/G0 cells (55% vs. 48%) and a 13% decrease in the number of S‐phase cells (20% vs. 23%) compared to HFD‐CM in MockT cells (Fig. 5E). Noteworthy, we see a decrease in the number of cells in G2/M in all voluntary PA groups compared to their sedentary counterpart in MockT cells (Fig. 5E). As with the protein changes we observed between HFD + LPA‐CM and HFD‐CM‐treated AdipoR1‐T cells, we found no difference in the number of cells in G0/G1 and S‐phase cells (Fig. 5F). By overexpressing AdipoR1 within the MCF7 cells, there again was a volume‐dependent effect of PA but to a lesser extent than in MockT cells. This may be due to the fact that the HFD‐CM effects were already blunted upon AdipoR1 overexpression (Fig. 5C vs. D).

## Discussion

It is now widely accepted that adipose tissue acts not only as an inert storage depot but as an active endocrine tissue via the production of adipokines which exert endocrine, paracrine and autocrine effects on the surrounding tissues and throughout the body. Although several hundred adipokines have been discovered to date, several studies have focused on ADIPO and LEP as they are the most abundant, have been shown to directly affect the growth of several cancers including breast and are altered in opposing fashion with obesity (Dieudonne et al. 2002, 2006; Zhong et al. 2010). Although ADIPO and LEP on their own have been shown to be associated with various cancers, emerging evidence now suggests that the ADIPO:LEP ratio may be a more reliable predictor (Chen et al. 2006; Ashizawa et al. 2010; Theriau et al. 2016). A hallmark characteristic of any cancer is genetic variability and instability, as such each cancer patient may possess a unique and specific carcinoma. This makes a broad tumor‐directed therapy between patients potentially costly and an imposingly difficult therapeutic direction. However, there are variables that can affect tumor growth that are more uniform across patients and regulated by stable components of patient physiology. One such characteristic is the overall growth microenvironment created by both the circulation and the adipose tissue surrounding a tumor. Alterations in the adipokine secretion profile in obese individuals may represent the molecular link between obesity and cancer. In breast cancer, this obesity‐cancer link has been clearly shown in postmenopausal women but the link is much less evident in premenopausal women.

In order to study the effects of obesity and adipose tissue expansion on breast cancer cell cycle regulation, we induced obesity in female rats using HFD‐feeding. We also evaluated the effects of PA as it has been shown to counteract obesity and alter the ADIPO:LEP ratio secreted by adipose in obese animals, both of which lower the incidence and severity of breast cancer (Zhu et al. 2008; Thompson et al. 2010; Malicka et al. 2015; Theriau et al. 2016). HFD‐feeding‐induced obesity in the animals (Fig. 1), and this altered the adipose tissue secretion levels of ADIPO and LEP, similar to results previously shown in humans and animals (Silha et al. 2003; Morad et al. 2014). PA increased the ADIPO:LEP ratio in both the serum and CM in a volume‐dependent manner (Fig. 2, Table 1) in agreement with previous published data in male rats (Thompson et al. 2010; Zhu et al. 2012; Theriau et al. 2016). Serum estradiol levels were positively correlated with obesity and serum LEP and inversely correlated with ADIPO, which also agrees with published results (Ollberding et al. 2013). These observations for the serum were not evident in adipose secretion of E2, as we observed no difference in estradiol in the CM between groups (Table 1). In female animals, PA was again able to abolish the HFD effects on MCF7 proliferation in a volume‐dependent manner as seen with male animals (Theriau et al. 2016). These volume‐dependent PA effects were evident in alterations in total body weight, body weight adjusted visceral fat mass (Fig. 1) and the subsequent ADIPO:LEP ratio both in serum and CM (Fig. 2; Table 1). These alterations led to changes in the tumor growth microenvironment that produced changes in MCF7 protein expression (increased pAMPK^T172^, p27, p27^T198^ and AdipoR1, decreased pAkt^T308^; Fig. 3), which ultimately resulted in overall accumulation of cells in the G1 phase of the cell cycle (Fig. 5). These effects of both diet and voluntary PA were similar to those previously shown in male animals, where estrogen levels are low (Theriau et al. 2016).

There has been mounting evidence for the strong association between obesity and breast cancer in postmenopausal women, but research is now suggesting that the greatest risk reduction in breast cancer due to regular PA occurs among premenopausal women (Friedenreich et al. 1998; McTiernan et al. 2003). Female rats in this study run a greater volume compared to male rats previously used (1.9‐fold increased km/day comparing HPA groups) which agrees with previously published data that female rats run more than males (Tokuyama et al. 1982; Eikelboom and Mills 1988). Although the animals ran differing volumes and adhered to higher PA cutoffs, we observed similar clear volume‐dependent effects of PA on altering the ADIPO:LEP ratio secreted by the adipose of HFD‐fed animals (Table 1). While we are unable to categorize the precise exercise performed (i.e. run, jog, walk), we show a linear effect of PA (distance run) on the adipose‐derived CM ADIPO:LEP ratio (Fig. 2C) and a volume‐dependent effect of PA on visceral fat and body mass in animals consuming an HFD. PA was able to ameliorate or abolish the effects of HFD‐CM depending on the volume of PA the animal engaged in, confirming the notion that PA represents a potential preventative and protective intervention for obese premenopausal women to reduce breast cancer proliferation. Thus, PA serves as a means to alter ADIPO:LEP ratio and subsequent tumor growth microenvironment in obese breast cancer patients, regardless of their menopausal status.

Interestingly, there was no difference between the effects of PA on the ADIPO:LEP ratio in female versus male animals (Theriau et al. 2016) regardless of the daily distance run. While females ran further both in the low and high PA groups, the overall trend and slopes of the regression lines were found to be almost identical. This indicates that the driving factor in altering the ADIPO:LEP ratio appears to be the volume or amount of PA an animal engages in and is not as reliant on circulating estrogen or diet the animal consumed. This strengthens the importance of PA in obese women in order to alter their ADIPO:LEP ratio and subsequent tumor growth microenvironment.

Although the overall trend was similar to that seen in male rats (Theriau et al. 2016), there were some distinct differences on the adipose‐dependent effects on the CM. We found the ADIPO:LEP ratio in the HFD‐CM using adipose from females and males was similar (128:1 vs. 122:1, respectively), however the ratio was 3.6‐fold higher in CD‐CM in female rats compared to male rats (2398:1 vs. 704:1, respectively). Therefore, if ADIPO and LEP are the primary contributors to the adipose‐derived tumor growth microenvironment, as previously suggested using male rats (Theriau et al. 2016), we would expect a greater effect of PA and chow diet on MCF7 cell function in this study. By comparing FACS results, we found that adipose tissue from female rats lessened the effects of HFD‐CM versus CD‐CM compared to CM prepared from male animals (Theriau et al. 2010). Although the ADIPO:LEP ratios were similar comparing HFD‐CM between studies, we found a 15% increase in the number of cells in G0/G1 and an 18% decrease in S‐phase in females. This lower proliferative effect of HFD‐CM on MCF7 growth may be due to estradiol effects on adipose adipokine secretion profile. This effect was also evident in all proteins examined. Using adipose from males, HFD‐CM decreased pAMPK^T172^, p27, p27^T198^, AdipoR1 and increased pAkt^T308^ further compared to CM prepared from female adipose (Theriau et al. 2016). This same relative pattern was also evident when comparing the effects of PA. In male animals, we found a 4.3‐fold increase in the ADIPO:LEP ratio between HFD + HPA‐CM and HFD + CM (Theriau et al. 2016). While in the female animals, we found the adipose‐derived ADIPO:LEP ratio to be 9.8‐fold higher in HFD + HPA‐CM compared to HFD‐CM. This translates to a 2.3‐fold increase in the ADIPO:LEP ratio between HFD + HPA‐CM and HFD + CM when comparing males and females, but this increased ADIPO:LEP ratio did not translate into greater G1 accumulation with PA in this study. We found similar alterations in cell‐cycle status comparing cells in G0/G1 (13% vs. 17% increase) and S‐phase (13% vs. 15% decrease) between HFD + HPA‐CM and HFD‐CM in females and males, respectively. Despite these observed effects on adipokine secretion, it remains clear that PA is an extremely effective intervention/prevention strategy for obesity‐linked cancers, regardless of whether estradiol is present or not. The ADIPO:LEP ratio was still found to be a predictor of the proliferative tumor growth microenvironment created by the adipose tissue. However, it appears that when estrogen levels are low (male rats), the change in ADIPO:LEP ratio required to effectively alter the adipose‐dependent growth environment are lower than those that would be necessary in the presence of higher levels of estrogen/E2 (female rats), as evidenced by lower overall associated cell‐cycle effects in female animals. Thus, there appear to be other primary effects of estrogen on adipokine secretion outside of those on ADIPO and LEP described herein.

We show the direct effects of adipose tissue and obesity to be blunted in female rats despite much larger changes in ADIPO:LEP ratio compared to male rats. This may point to adipokine secretion profile as being one of the plausible mechanisms underlying the less reproducible link between obesity and breast cancer in premenopausal women compared to postmenopausal women. Given the complexity of the adipose secretome, it is highly likely that estrogen alters the secretion of adipokines other than ADIPO and LEP as previously shown (Machinal et al. 1999; Morad et al. 2014), affecting the complete adipose‐dependent effects on tumor growth microenvironment. Certain adipokines and inflammatory cytokines have been shown to be decreased by estrogen or in premenopausal compared to postmenopausal women, are altered with obesity, have been shown to affect the tumor growth microenvironment and proliferation of breast cancer cells such as resistin (Savage et al. 2001; Huang et al. 2005; Kim et al. 2011; Assiri et al. 2015). One such possible adipokine could be resistin, since resistin accelerates S‐phase entry and its secretion from adipose is repressed by estrogen (Silha et al. 2003; Huang et al. 2005). This may help to explain the blunting we see in female compared to male animals. In males, there would be greater additive cell cycle entry effects of resistin and LEP promoting proliferation compared to in females where estrogen‐dependent reductions in resistin would be evident, lessening the cumulative effects of resisting and LEP.

Independently of the adipose contribution to breast cancer growth, there exists a distinct benefit of stabilizing AdipoR1 signaling in breast cancer cells. Previous work in our lab has shown stably overexpressing AdipoR1 can enhance the effects of ADIPO, counteracting the effects of HFD on adipose‐dependent alterations in tumor growth environment (Theriau et al. 2016). This is important as research has shown AdipoR1 protein levels are decreased in the visceral adipose tissue of obese women (Rasmussen et al. 2006), downregulated in preinvasive ductal carcinoma in‐situ (Pfeiler et al. 2010) and LEP has been shown to down regulate AdipoR1 mRNA in breast cancer cells (Jarde et al. 2009). We found that overexpressing AdipoR1 was able to abolish the effects of the HFD‐CM on all cell‐cycle proteins measured (Figs 3 vs. 4) as well as overall cell‐cycle status (Fig. 5C vs. D). This strengthens the notion that regardless of the sex of the animal used to create adipose‐derived CM, increasing AdipoR1 protein levels increases antiproliferative effects of ADIPO, thereby suppressing tumor growth. This highlights AdipoR1 as a target for novel breast cancer pharmacological therapeutics regardless of estrogen or menopausal status.

Cumulatively, our results highlight the importance of adipose tissue in controlling the tumor growth microenvironment surrounding breast cancer cells. We show that the adipose tissue appears to have a greater effect on controlling the tumor growth microenvironment in male rats, where estrogen levels are low (Theriau et al. 2016), compared to female rats where circulating estrogen is elevated, altering the adipokine secretion profile and subsequent growth environment a breast cancer is exposed to. This provides a potential mechanism explaining why obesity may not have as strong of an observed epidemiological association on tumor growth in premenopausal women as compared to postmenopausal women (Kaaks et al. 1998; Lahmann et al. 2004; Cecchini et al. 2012). Furthermore, when isolating the effects of the adipose alone we do not observe any of the previously published “protective” effects of obesity on breast cancer (Ursin et al. 1995; van den Brandt et al. 2000; Michels et al. 2006). Regardless of estrogen status or gender, PA can counteract the effects of “obese” adipose tissue on promoting breast cancer cell proliferation. AdipoR1 plays an important role in the regulation of MCF7 cell growth and stabilization of the receptor, especially in an obese background, and presents a possible method of slowing obesity‐dependent breast cancer cell growth. Although our work used ADIPO and LEP as markers of adipokine secretion profile, we are in no way suggesting that these are the only adipokines produced by adipocytes that underlie adipose‐dependent effects. In fact, for ovulating female rats there appears to be a more evident contribution of these other adipokines. Estrogen appears to alter the adipose tissue, affecting the growth microenvironment that a breast cancer is exposed to. However, it is clear that the ADIPO:LEP ratio still displays a strong representation for accurately predicting the growth microenvironment that a breast cancer in an obese patient is exposed to in both post‐ and premenopausal women. Therefore, therapies that alter the levels/ratio of these adipokines or act to increase the expression of the AdipoR1 may represent interventions that can alter tumor growth microenvironment, increasing the chance of success in obese breast cancer patients regardless of menopausal status.

## Conflict of Interest

None declared.
